# Laterality in the Damaraland Mole-Rat: Insights from a Eusocial Mammal

**DOI:** 10.3390/ani13040627

**Published:** 2023-02-10

**Authors:** Paul J. Jacobs, Maria K. Oosthuizen

**Affiliations:** 1 Department of Zoology & Entomology, University of Pretoria, Private Bag X20, Pretoria 0028, South Africa; 2 Mammal Research Institute, University of Pretoria, Pretoria 0002, South Africa

**Keywords:** laterality, behavioral asymmetry, Damaraland mole rat, sociality, eusocial, turning biases, captivity

## Abstract

**Simple Summary:**

Side biases observed in behavior are thought to reflect underlying asymmetric brain function or hemispheric specialization. These asymmetries occur at the individual and population level, although population-level laterality normally is only evident in social species. In a previous study, we found both individual- and population-level laterality in a solitary mole rat species. Here, we assessed laterality in a eusocial mole rat species, the Damaraland mole rat, *Fukomys damarensis*, using turning biases. All individuals combined demonstrated left-turning biases, which was also significant at the population level. Wild-caught animals were more strongly lateralized, but lacked the population-level left-turning bias that was observed in captive mole rats. This emphasizes the importance of context and animal handling when measuring and interpreting behavioral asymmetries.

**Abstract:**

Lateralization is the functional control of certain behaviors in the brain being processed by either the left or right hemisphere. Behavioral asymmetries can occur at an individual and population level, although population-level lateralization is less common amongst solitary species, whereas social species can benefit more from aligning and coordinating their activities. We assessed laterality (individual and population) through turning biases in the eusocial Damaraland mole rat, *Fukomys damarensis.* We considered factors such as breeding status (queen or subordinate), environment (wild-caught or captive), sex (male or female), colony and body mass. All individuals together demonstrated significant left-turning biases, which was also significant at the population level. Wild-caught animals were more strongly lateralized, had a wider spread over a laterality index and lacked the population-level left-turning bias as compared to captive mole rats. Subordinate animals were more lateralized than queens, demonstrating social status differences in turning biases for social mole rats. This emphasizes the importance of animal handling and context when measuring and interpreting behavioral asymmetries.

## 1. Introduction

It is now well accepted that most vertebrates have strong left–right asymmetries in their brains and in their behaviors [1,2,3,4,5]. These asymmetries cause biases in the processing of specific stimuli [6,7,8]. Lateralization can be observed as specific behavioral inclinations such as motor biases, including turning or “handedness” [8,9,10,11], or sensory biases such as nostril use in horses [12] or eye use in chicks [13]. Each hemisphere controls specific behaviors, where the left hemisphere controls sustained responses to targets, whereas the right hemisphere is used for the control of potent releasers of innate responses [14]. The differential use of one hemisphere over the other can indicate preferential cognitive and emotional processes, revealing specific information about animal behavior [7,15,16,17]—for example, aggressiveness and fearfulness in right-hemisphere-dominant animals [10,16] and boldness in left-hemisphere-dominant animals [18]. It is therefore beneficial to avoid the duplication of function in the hemispheres, and this is supported in a variety vertebrates and invertebrates [14,19,20,21]. The laterality of an organism is likely the result of the interaction between genetic and environmental drivers [21,22], where factors such as light, hormones, rearing environment, pollution and stress have been determined to influence the development of the lateralized brain [4].

Interestingly, behavioral asymmetries can arise at an individual and a population level [19,23]. Individual laterality refers to the number of left- and right-biased individuals in a population where most individual are lateralized, which can often occur roughly half in one direction and the other half in the other direction [14,17]. These biases can vary in strength among individuals of the same species, or different species, and also depends on the task considered [14,24]. Lateralized behavior at the individual level is predicted to have positive and negative effects [10,14,19]. For instance, in terms of cognitive abilities, lateralized individuals have demonstrated increased numerical competence [6,25], stronger discrimination performance [26] and even predator detection [27]. However, there are cases where unlateralized function of the brain can be beneficial—for example, more lateralized individuals presented greater stress responses in Port Jackson sharks (*Heterodontus portusjacksoni*) [28] and lambs [29], and lateralized individuals are more likely to attack conspecifics in fish [11], all factors that are beneficial for animals kept in captivity. Overall, laterality is believed to provide a net fitness benefit to an individual, but it does not explain population-level laterality [19,30].

Population-level laterality refers to the condition where most individuals from a population have the same lateralized one directional bias—for example, human handedness [14,19,24]. It has been proposed that when individually asymmetric organisms must interact with conspecifics and coordinate their activities, asymmetry aligns in the majority of individuals in a population [17,20,30]. This suggests that social species are more likely to show population-level asymmetries, as social species are more likely to interact and coordinate socially [24,31,32]. In turn, members within a social group may experience greater fitness benefits when performing the same behavioral stratagem [17]. Currently, the evolution of individual lateralization does not predict any population-level lateralization [20]. It is possible that processes involving individual laterality do not influence population-level laterality if not relevant in a social context. In addition, if population-level asymmetries are present in solitary species, they should be less strongly lateralized or absent as opposed to species that interact socially [20,30].

Comparisons of solitary and social lateralized behavior have been made in bees. The solitary mason bee (*Osmia cornuta*) displays population-level asymmetries when involved in social agonistic encounters [33]. However, during an olfactory memory experiment, population-level asymmetries were absent in the solitary bee, but not in the eusocial honeybee (*Apis meliffera*) [34]. These results suggest that population-level asymmetries only manifest under social conditions. Nevertheless, in the solitary Cape mole rat (*Georychus capensis*), a significant left-turning population bias was observed when the laterality index (LI) scores of all individuals were compared [35]. This is one of the few studies demonstrating a population-level asymmetry in a solitary species in a non-social context and contradicts the current notion that population-level asymmetry is only present in social contexts [17,20,30].

African mole rats exhibit a wide social spectrum, ranging from solitary to eusocial [36,37], where eusociality is the most extreme case of social behavior [38]. Social mole rats display a reproductive division of labor and cooperative care of the young, which extend over at least two generations [38,39,40]. Inclusive fitness of this reproductive structure results in minimal internal competition and highly cooperative behavior [41,42,43,44] and therefore the alignment of population laterality in cooperative behavior is expected to be high [17]. To date, eusociality has only been described in two mammals, namely the naked mole rat (*Heterocephalus glaber*) and the Damaraland mole rat (*Fukomys damarensis*) [37,45,46]. These two mole rat species display all facets of eusociality, in addition to Michener’s [47] definition of sociality, which includes overlapping generations, cooperative brood care and reproductive division of labor. They also have a high reproductive skew where the maximum lifetime fecundity of breeders versus non-breeders (helpers) is far greater than that of female breeders versus helpers in other cooperatively breeding, vertebrate societies, as outlined by Sherman et al. [48].

The Damaraland mole rat, being a eusocial species, is therefore an ideal model species to investigate behavioral asymmetry. Firstly, it allows for eusocial to solitary lateralized comparison, as done in bees [33,34], but also allows us to investigate eusocial and solitary lateralized comparisons in mammals. In this study, we investigated individual- and population-level laterality in the Damaraland mole rat, under the same experimental conditions as was performed for the Cape mole rat [35]. It is important to note that turning biases were used, which is considered to be an individual-level laterality trait [9]. We predict that population-level asymmetry should be present in the Damaraland mole rat, and that the strength of the directional bias should be higher than in the Cape mole rat, as suggested by the evolutionary stable strategy (ESS) for social and non-social species hypothesis [17,20,30]. Environmental conditions (captive or free-living) may be important as predator responses are absent in captivity, and could further promote a left-hemisphere-dominant process if exposure to stressful situations persists [10]. Additionally, the study by Jacobs and Oosthuizen [35] on a solitary mole rat revealed that wild-caught and captive individuals differed in the strength of laterality. Thus, we anticipate that wild-caught Damaraland mole rats will demonstrate higher directional strength in laterality to be overall more lateralized in a particular direction compared to captive animals. Lastly, we expect that a left-turning bias will be observed in the Damaraland mole rats, similar to what was seen in the Cape mole rat by Jacobs and Oosthuizen [35].

## 2. Material and Methods

### 2.1. Data Acquisition

The current study utilized data obtained from recorded videos from another project that investigated the learning and memory of Damaraland mole rats [49]; thus, the experimental design was determined by that experiment.

### 2.2. Animal Capture and Husbandry

In order to determine whether queens differed from subordinates in laterality and to test for the effect of the colony, only groups consisting of at least one queen and one subordinate were used. Thus, sample sizes differed between this study and the study by Oosthuizen [49]. The experimental animals originated from laboratory colonies at the University of Pretoria, and all were born in captivity. Although Damaraland mole rats can live in captivity for up to 16 years (Oosthuizen, pers. obs.), animals in the study were less than 10 years old and considered healthy adults. Queens are generally the older individuals in the colony, but the ages of the different colonies may vary [46,50]. The experimental group consisted of 15 animals (5 queens and 10 subordinates (3F and 7M). Animals from the field group were captured near Blackrock (27°7′ S, 22°52′ E) in the Northern Cape, South Africa, using Hickman live traps [51]. This group was supplemented with animals from Tswalu Kalahari Reserve (27°31′ S, 22°19′ E), Northern Cape; these animals were subsequently released again. The field group consisted of 25 animals (6 queens and 19 subordinates (10F; 9M)). All animals were housed in plastic crates within their respective colonies. Crates were lined with wood shavings and animals were provided with tissue paper for nesting material. Animals were fed ad libitum on chopped sweet potatoes, apples and carrots, and they retrieved their water from the food. Trapping permits were obtained from the Northern Cape Nature Conservation Authority (ODB 2023/2010) and experimental procedures were approved by the Animal Use and Care Committee at the University of Pretoria (EC013-09).

### 2.3. Experimental Design

**Figure 1:**
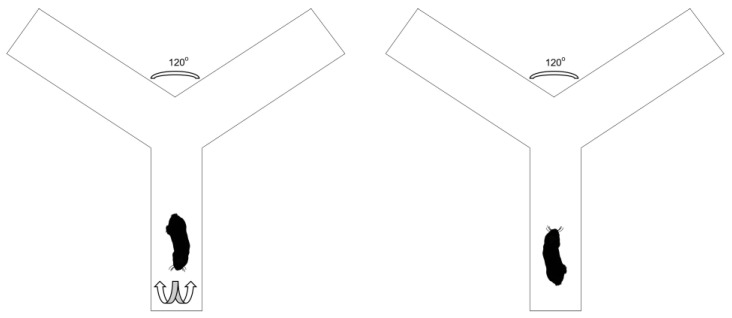
The setup of the Perspex Y-maze, and how individual Damaraland mole rats were placed into the Y-maze facing away from the center and had to make a directional turn in order to reach the middle of the maze. The direction of the turn to reach the middle of the maze was used as the turning bias.

A Perspex Y-maze (arms 50 cm × 10 cm × 20 cm) was used as a testing apparatus. Two of the arms of the Y-maze were blocked on the distal ends, while the third one was attached to a nest box. Animals were released in one of the blocked arms, facing away from the center, and the direction of the first turn that the animals made towards the middle of the maze was recorded to determine the laterality of the animals (Figure 1). The experiment was filmed with an overhead video camera and analyzed upon completion of the experiment. The video footage of the animals was used to log the direction in which the animals turned. The turn at the Y-section of the maze was not used and did not contribute to turning bias determination. Each animal was subjected to 10 trials per day for four consecutive days. The time between trials varied between 45 and 60 min.

### 2.4. Determination of Turning Biases, Laterality Index and Absolute Laterality

If an initial turn could not be determined due to complications of placing a mole rat into the maze, then the turn was discarded, and the total number of turns was reduced. The minimum number of turns was 35, and demonstrated individual turning biases (Table 1). The laterality index (LI) was determined for each mole rat by computing the total number of left turns across all trials. We used the following formula: (number of left turns–number of right turns)/(total number of turns (40 in this case) × 100). This provides a measure of the population-level asymmetry [21,52,53]. Absolute laterality (AL) is the absolute value of the LI and represents the strength of the laterality irrespective of their preferences to turn left or right [21,53]. AL values were converted to a percentage, and thus the AL index ranges from 0 (an individual that turned in equal proportion to the right and to the left—no bias) to 100 (an individual that turned in the same direction in all trials).

### 2.5. Statistical Analysis

All statistical analyses were performed in R 4.2.1 [54]. Each individual mole rat was tested for laterality regarding their turning biases by comparing the number of left turns to right turns in a non-parametric two-tailed binomial test. The response variables, the LI and AL scores were tested for normality using the Shapiro–Wilk test. AL scores were not normally distributed. Homogeneity of response variables were confirmed with Levene’s test. To determine if there was an inherent turning bias or if turning bias was random in Damaraland mole rats, the LI scores of all individuals were compared in a one-tailed *t*-test, first pooled together and again separately for wild and captive individuals to make it comparable to the Cape mole rat [35]. In order to determine whether LI and AL were influenced by colony, body mass, sex (male or female), housing conditions (wild-caught or captive) and breeding status (queens or non-breeding subordinates), a linear model for LI and a general linear model using a gamma log-link function for AL were used. Data were analyzed using a linear model using the *lme4* package [55]. Backwards elimination of linear models was performed using the *step* function of the lmerTest package in order to determine the best model for each response variable, determined through the AIC criterion [56]. Significant variables in the regression models were followed up with post hoc comparisons, conducted using Tukey’s HSD pairwise comparisons using the *emmeans* package [57] (Figures S1 and S2). Data are presented as mean ± standard error (s.e.m), and a *p*-value of ≤0.05 was defined as significant.

## 3. Results

For all analyses, we only report the results of the best model based on the AIC criterion. LI followed a normal distribution (W = 0.96, *p* = 0.19) and did not violate homogeneity (F = 0.42, *p* = 0.82). AL was not normally distributed (W = 0.85, *p* < 0.0001), but did not violate assumptions of homogeneity (F = 0.62, *p* = 0.68).

### 3.1. Individual Turning Biases

There was no difference in turning biases between the wild group (9/25: 36%) and the captive group (5/15: 33%) (Table 1).

### 3.2. Individual Laterality, Population-Level Laterality and Strength of Direction of Laterality

Overall, a population-level left-turning bias was observed when all mole rats were grouped together (LI for all mole rats: one-sample *t*-test = 2.2813, df = 39, *p* = 0.03). When investigating whether captivity influenced the overall turning bias, the captive population was found to have a significant left-turning bias (LI for captive mole rats: one-sample *t*-test = 2.6688, df = 14, *p* = 0.02), whereas the wild population did not (LI for wild-caught mole rats: one-sample *t*-test = 1.112, df = 24, *p* = 0.28).

The best linear model for LI (directional bias and population laterality) showed that only body mass, captivity and breeding status and breeding status × captivity influenced laterality, but no specific variable contributed significantly to the variation in LI (R^2^ = 0.05498, F = 1.567 (4,35), *p* = 0.20).

**Figure 2:**
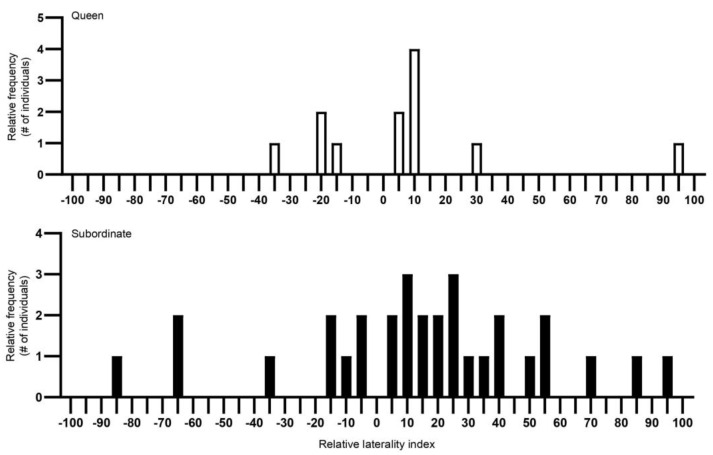
The relative frequency (Y-axis) of Damaraland mole rat (*Fukomys damarensis*) queens’ (*n* = 11) and non-reproductive subordinates’ (*n* = 29) individual laterality along a laterality index (X-axis). The Y-axis scale represents the frequency of individuals with the same lateral index. The X-axis scale from the left represents right turning, with increased turning to the left as the index increased from −100 to 100. A laterality index value of 0 means that an individual turned left and right in equal amounts, demonstrating no turning biases. White bars represent queens and black bars represent subordinates. #—number.

**Figure 3:**
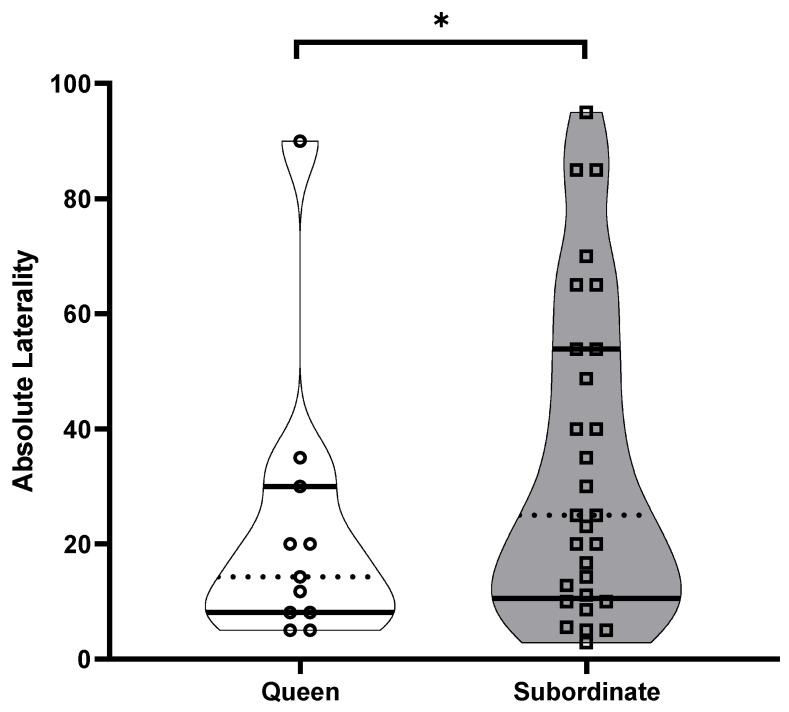
The absolute laterality (directional strength in laterality) in the Damaraland mole rat *Fukomys damarensis* between queens (open circles: *n* = 11) and non-reproductive subordinates (open squares: *n* = 29). The dashed line represents the median, with solid lines representing the upper and lower quartiles. * indicates significance at *p* < 0.05.

**Figure 4:**
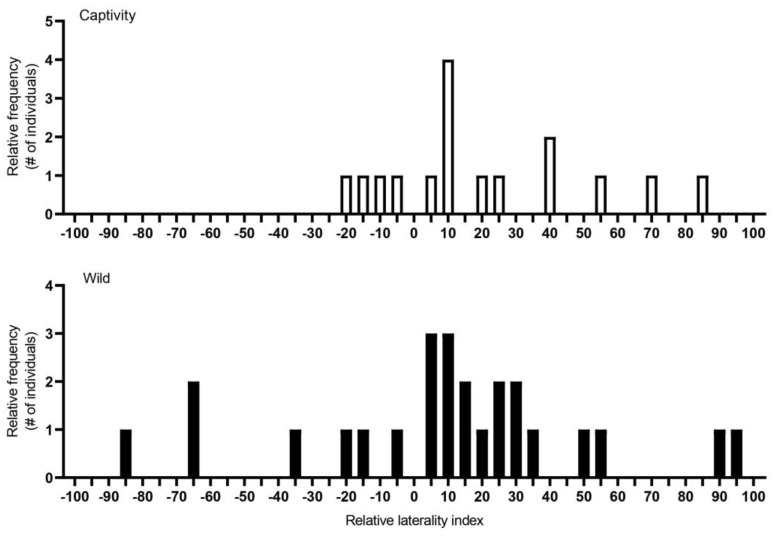
The relative frequency (Y-axis) of captive (*n* = 15) and wild-caught (*n* = 25) Damaraland mole rats’ (*Fukomys damarensis*) individual laterality along a laterality index (X-axis). The Y-axis scale represents the frequency of individuals with the same lateral index. The X-axis scale from the left represents right turning, with increased turning to the left as the index increased from −100 to 100. A laterality index of 0 means that an individual turned left and right in equal amounts, demonstrating no turning biases. Black bars represent wild-caught animals and white bars represent animals in captivity. #—number.

**Figure 5:**
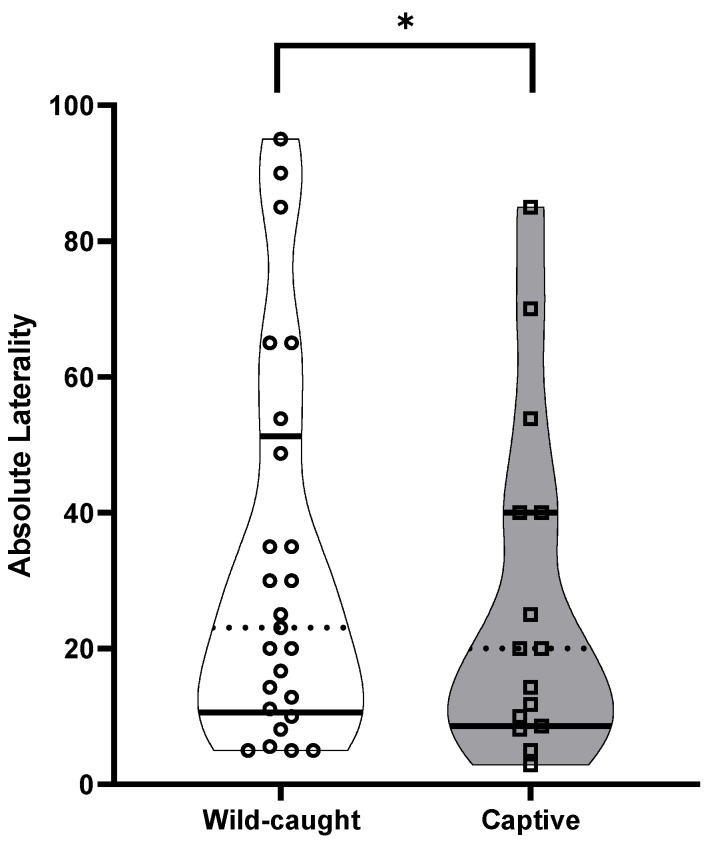
The absolute laterality (directional strength in laterality) in the Damaraland mole rat *Fukomys damarensis* between wild-caught (open circles: *n* = 25) and captive animals (open squares: *n* = 15). The dashed line represents the median, with solid lines representing the upper and lower quartiles. * indicates significance at *p* < 0.05.

The best general linear model for AL (strength of the bias) showed that colony (t = −5.741, *p* < 0.001), breeding status (t = 2.241, *p* = 0.03) and captivity (t = −2.520, *p* = 0.02) each contributed significantly to the variation in AL. Post hoc analyses revealed that queens (AL: 22.48 ± 7.38) and subordinates (AL: 33.84 ± 5.06) significantly differed in the strength of the bias, where subordinates demonstrated stronger directional biases compared to queens (t = 2.241, *p* = 0.03) (Figure 2 and Figure 3). Post hoc analyses for captivity show that wild-caught individuals (AL: 32.58 ± 5.60) more often had a stronger directional bias compared to captive individuals (27.64 ± 6.48) (t = −2.520, *p* = 0.02) (Figure 4 and Figure 5).

## 4. Discussion

It has been proposed that population-level laterality likely arises only under the pretext of social selection pressures [17,20,24,30]. A study performed by Jacobs and Oosthuizen [35] found that a solitary mole rat species had a population-level left-turning bias. This contradicts the hypothesis of the social coordination of laterality (SCL). In this study, we used a eusocial mole rat, the Damaraland mole rat, to determine how a social mole rat may differ from a solitary mole rat. We expected some lateralized processes at a population level from the social species, as similar asymmetries may contribute to social interactions, cooperative behavior and colony function [16,58,59]. Similar to the solitary Cape mole rat, the social Damaraland mole rats demonstrated a population-level left-turning bias when all individuals were collectively analyzed. This reflects the dominance of the right hemisphere, and is congruent with our original prediction [35]. Interestingly, specific factors such as colony, body mass, sex and breeding status (queen or subordinate) were not associated with this population-level asymmetry. Furthermore, individual laterality differed when Damaraland mole rats were separated into captive and wild-caught populations. We found a significant difference in the strength of laterality between wild-caught and captive Damaraland mole rats, with a similar but not significant pattern observed in the solitary mole rat [35]. Only captive individuals retained their population-level left-turning bias, which was absent in the wild-caught population.

Colony affiliation, body mass, sex and breeding status did not significantly determine population-level laterality and may suggest that other factors that were not measured in the current study may preclude the observed population bias. The most likely cues used for turning biases may be sensory (olfactory [60], visual [61], tactile [62] or auditory [63]). However, some of these cues may not be relevant in this context due to their limited use in a subterranean environment. It is unlikely that Damaraland mole rats used these cues in a social sense in this current setting, as individuals were measured individually; however, this does not imply that inherent biases due to social factors are not present. One potential reason for the observed turning bias is a predator response, despite predation being more likely to occur during dispersal when animals are not protected by their subterranean niche [37,64]. Other confounding cues that were controlled for included the orientation of the maze, as mole rats may orientate themselves based on a magnetic compass [65]. Learning also would not affect turning biases, as the initial turn measured had no reward associated with it, eliminating confounding effects of dopamine on behavioral preferences [66]. Furthermore, the observed turning behavior was not influenced by thigmotaxis as individuals turned without touching the walls of the maze [67].

Captive and wild-caught mole rats are exposed to vastly different environmental conditions, and the differential exposure to atmospheric conditions (hyperoxia vs. hypoxia) could potentially affect their development [36,37]. Studies on fish and rats have demonstrated behavioral asymmetrical changes in animals exposed to changes in environmental conditions [68,69]; however, it is uncertain to what extent this type of phenomenon may affect mole rats. Due to the limited comparisons of individuals within the same group, and subordinates to queens, colonies should be investigated further as single subordinate to queen comparisons may have skewed the colony-level behavioral asymmetry. Future studies should strive for a larger sample size of individuals from the same colony and compare queens with the remainder of the colony, as well as comparing queens from different colonies. It is important to note that turning biases are motor skills in a non-social context; however, when considered for cooperative foraging, it may be relevant for the emergence of population-level laterality. This could suggest that although turning biases were used to determine population-level laterality in the social and solitary species, the mechanisms that underlie the biases may differ. It will be interesting to measure population-level laterality in the solitary and social mole rats together utilizing social cues, as was done in the mason bee and honey bee comparisons [34,59].

Rogers et al. [70] predicted that captive-bred and wild- caught populations may differ because they would be subjected to different genetic selection, as well as differing environmental influences on development. Several other comparisons between individuals from the wild and captive populations have been made, such as the manatee [71], parrots and cockatoos [21,72], chimpanzees [73], ayes-ayes [74] and rainbowfish [75], where no significant differences were found between wild and captive populations. This is in contrast to the current study, as a significant difference was observed between wild-caught and captive populations for the strength of laterality. One additional factor present in our study but absent in the others is the handling of animals while they were transferred during experimentation. Handling of the animals induces behaviors mediated by the right hemisphere, such as aggression [33,76,77,78,79,80] and stress [10,28,29,81,82], both of which can differ within wild-caught and captive individuals. Since the experimental design in the current study and the study by Jacobs and Oosthuizen [35] was identical, it is likely that the handling and the transferring of animals into the maze may have initiated a self-defense aggressive response from animals. Interestingly, agonistic interactions resulted in population-level asymmetries in a solitary species [59]. It is important to note that mole rats are generally very xenophobic and aggressive, but solitary species are more so [35,83,84]. This may suggest that aggression towards being handled, and not general aggression, may be relevant here and requires an experimental design to avoid handling and alternative means of determining behavioral asymmetry.

It has been proposed that captive individuals may benefit from being left-hemisphere-dominant due to their reduced aggression [8,10,17]. Captive Damaraland mole rats demonstrated reduced behavioral asymmetry in turning biases; however, it was still a right-hemisphere-dominant process, similar to what was observed in the Cape mole rat [35]. This suggests that despite the enrichment of a social lifestyle in captivity, negative emotional biases are still present for social mole rats in captivity [10]. Therefore, our study supports the notion that merely being social is not enough for the enrichment of animals to prevent negative emotional biases.

The relation of functional cerebral asymmetries and stress (measured as a glucocorticoid stress response) [85,86,87,88] has been investigated in several different species (reviewed by Ocklenburg et al. [81]). Stressed animals rely predominantly on the use of the right hemisphere [10,89,90,91]. Previous comparisons of glucocorticoids in Damaraland mole rats revealed similar glucocorticoid levels between subordinates and the queen in a colony; however, as a group, captive Damaraland mole rats have higher glucocorticoid levels than wild animals [92]. Stress differences are also apparent between solitary and social African mole rat species [93], where solitary mole rats have much higher stress cortisol levels as compared to social mole rats [93]. Differences in the behavioral and physiological responses of social and solitary mole rats to handling, and the resulting aggression and/or stress in response to an experimental handler, may be two critical factors that can predispose mole rats to negative emotional bias of the right hemisphere [10,14,28,81]. This may explain the population-level asymmetry that was observed in the current study, and in the Cape mole rat by Jacobs and Oosthuizen [35].

Damaraland mole rat queens were found to be less lateralized than subordinates, and since stress levels are similar between subordinates and queens, it does not explain why queens and subordinates differed in the strength of directional biases [10,28,81,92]. Several aspects of the Damaraland mole rat queen physiology, ecology and morphology may contribute to behavioral asymmetrical changes later in life [37,50,94,95,96,97]. Although most behavioral asymmetrical changes are a result of neonatal changes [31], it is not known to what extent changes associated with queen succession may affect behavioral asymmetry—for example, activational endocrine-related changes that may persist permanently [98,99]. Age-related changes may also be apparent in behavioral asymmetry. Age-related asymmetry reduction is predicted for older individuals [100,101,102,103,104] and could be relevant to our current findings, as queens are generally older than other colony members [37,105]. Importantly, social mole rats utilize reproductive suppression, which results in a reduced GnRH response in non-reproductive individuals [106,107,108] and queens having a different neuroendocrine phenotype [109]. These are some factors that separate queens from subordinates and may also result in the observed differences in behavioral asymmetry between them.

## 5. Conclusions

We found a population-level left-turning bias when all Damaraland mole rats were collectively analyzed, but this significance disappeared once captive and wild-captured Damaraland mole rats were analyzed separately. Captive mole rats had a lower directional strength in laterality compared to wild-caught individuals, but the population-level left-turning bias was maintained in the captive population, and was lost in the wild-caught individuals. We suggest that handling during experimentation can cause stress and aggression, resulting in agonistic interactions, and may have resulted in a left-turning bias regardless of captive or wild-caught status. We propose that experimental contexts, which may bias stress and aggression, are important factors that may give rise to population-level asymmetries even under non-social contexts. Our study considered only turning biases, but in order to determine whether the population-level asymmetries observed here were not due to stress and aggression, other social and non-social motor and/or sensory lateral processes that are devoid of agonistic and/or stressful situations should be investigated. One should also consider how captive and wild-caught populations may differ once handler-induced stress and/or aggression is absent.

## Figures and Tables

**Table 1:** Damaraland mole rat (*Fukomys damarensis*) queen and non-breeding subordinate left- or right-turning bias preference from wild-caught and captive populations within their respective colonies. For each individual, the total numbers of left and right turns are given with the total number of trials, together with the two-tailed binomial significance (*p* or ns for not significant). Significance of the binomial probability demonstrates that an individual is lateralized.

Mole #	Colony	Wild/Captive	Breeding Status	Left Turns	Right Turns	Total Turns	Binomial Probability
WB1	1	Wild	Queen	38	2	40	*p* < 0.05
WB2	1	Wild	Subordinate	7	33	40	*p* < 0.05
WB3	2	Wild	Queen	13	27	40	*p* = 0.04
WB4	2	Wild	Subordinate	7	33	40	*p* < 0.05
WB5	2	Wild	Subordinate	19	21	40	ns
WB6	3	Wild	Queen	16	24	40	ns
WB7	3	Wild	Subordinate	21	19	40	ns
WB8	3	Wild	Subordinate	39	1	40	*p* < 0.05
WB9	3	Wild	Subordinate	3	37	40	*p* < 0.05
WT1	8	Wild	Subordinate	24	15	39	ns
WT2	8	Wild	Subordinate	27	13	40	*p* = 0.04
WT3	8	Wild	Subordinate	26	14	40	ns
WT4	8	Wild	Subordinate	22	18	40	ns
WT5	8	Wild	Queen	26	14	40	ns
WT6	9	Wild	Subordinate	30	9	39	*p* < 0.05
WT7	9	Wild	Queen	21	19	40	ns
WT8	9	Wild	Subordinate	25	15	40	ns
WT9	9	Wild	Subordinate	22	17	39	ns
WT10	9	Wild	Subordinate	24	16	40	ns
WT11	9	Wild	Subordinate	29	10	39	*p* < 0.05
LE1	16	Wild	Subordinate	21	15	36	ns
LE2	16	Wild	Subordinate	20	16	36	ns
LE3	16	Wild	Subordinate	19	17	36	ns
LE4	16	Wild	Subordinate	15	20	35	ns
LE5	16	Wild	Subordinate	20	17	37	ns
L9	11	Captive	Queen	21	19	40	ns
L10	11	Captive	Subordinate	24	16	40	ns
L11	11	Captive	Subordinate	30	9	39	*p* < 0.05
L12	11	Captive	Subordinate	37	3	40	*p* < 0.05
L13	12	Captive	Queen	16	24	40	ns
L14	12	Captive	Subordinate	34	6	40	*p* < 0.05
L15	12	Captive	Subordinate	25	15	40	ns
L16	12	Captive	Subordinate	18	22	40	ns
L17	12	Captive	Subordinate	28	12	40	*p* = 0.02
L18	13	Captive	Queen	19	15	34	ns
L19	13	Captive	Subordinate	28	12	40	*p* = 0.02
LE13	19	Captive	Queen	20	17	37	ns
LE14	19	Captive	Subordinate	17	18	35	ns
LE15	20	Captive	Subordinate	19	16	35	ns
LE16	20	Captive	Queen	15	20	35	ns

Note: All subordinates are considered non-breeders. Mole # WB and WT were wild animals from Black Rock and Tswalu and # L and LE were laboratory animals. Significance is at *p* < 0.05.

## Data Availability

Data is contained within the article.

## Supplementary Materials

The following supporting information can be downloaded at: https://www.mdpi.com/article/10.3390/ani13040627/s1, Figure S1: The emmeans comparisons of absolute laterality (absolute value of the laterality index) between queens and subordinates (SUB), where arrows represent comparisons, and arrows which do not overlap represent significant comparisons; Figure S2: The emmeans comparisons of absolute laterality (absolute value of the laterality index) between wild-caught (W) and captive (C) individuals, where arrows represent comparisons, and arrows which do not overlap represent significant comparisons.
