# Supplementary material for: Laterality in the Damaraland Mole-Rat: Insights from a Eusocial Mammal
Source: Animals (Basel). 2023 Feb 10;13(4):627. doi: 10.3390/ani13040627 (PMC9951763; doi:10.3390/ani13040627)
Supplement: Supplementary file 1 [file animals-13-00627-s001.zip › animals-2177085-supplementary.pdf]

animals

MDPI

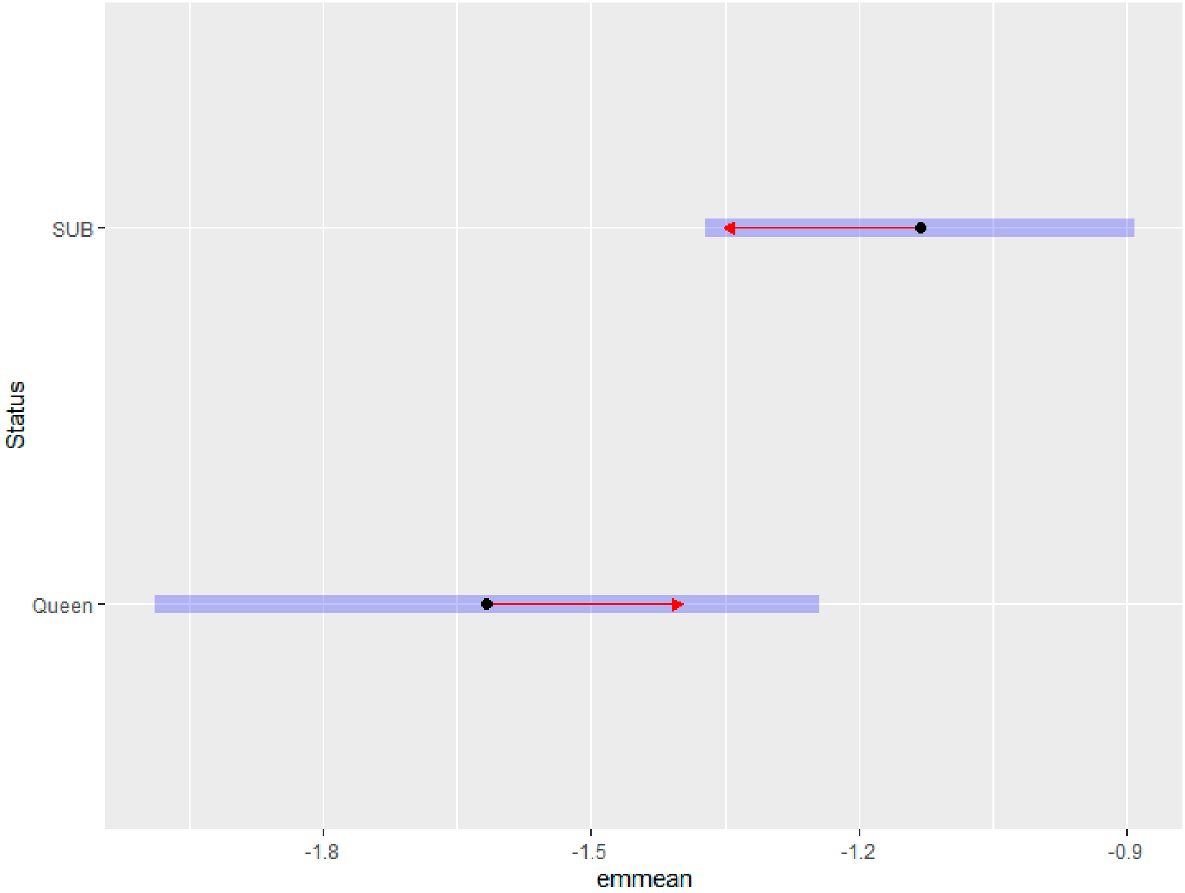

Figure S1. The emmeans comparisons of absolute laterality (absolute value of the laterality index) between queens and subordinates (SUB), where arrows represent comparisons, and arrows which do not overlap represent significant comparisons.

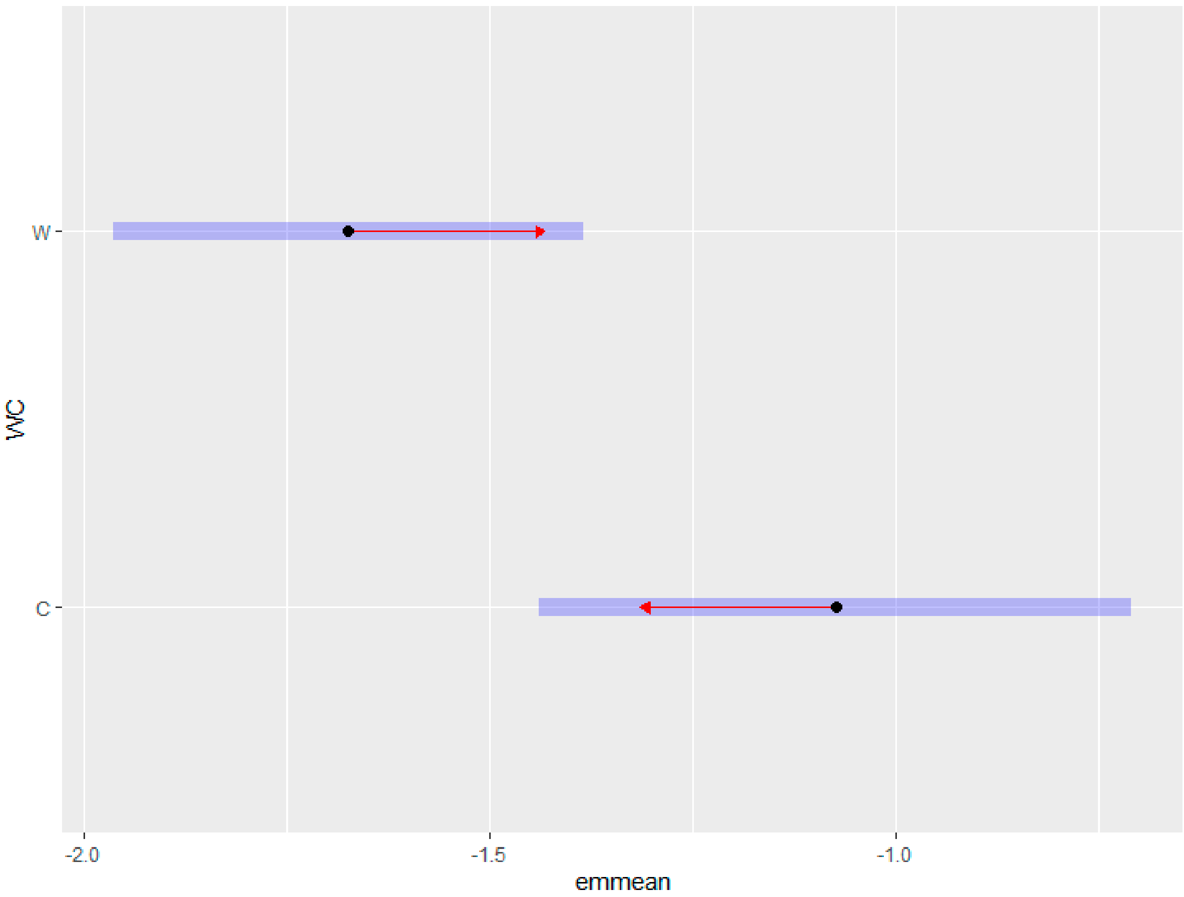

Figure S2. The emmeans comparisons of absolute laterality (absolute value of the laterality index) between wild-caught (W) and captive (C) individuals, where arrows represent comparisons, and arrows which do not overlap represent significant comparisons.
